# Uncovering co-expression gene network modules regulating fruit acidity in diverse apples

**DOI:** 10.1186/s12864-015-1816-6

**Published:** 2015-08-16

**Authors:** Yang Bai, Laura Dougherty, Lailiang Cheng, Gan-Yuan Zhong, Kenong Xu

**Affiliations:** Horticulture Section, School of Integrative Plant Science, Cornell University, New York State Agricultural Experiment Station, Geneva, NY 14456 USA; Horticulture Section, School of Integrative Plant Science, Cornell University, Ithaca, NY 14853 USA; USDA-ARS, Plant Genetic resource and Grape Genetic Research Units, Geneva, NY 14456 USA

**Keywords:** *Malus*, Fruit acidity, *Ma1*, Aluminum-activated malate transporter1 (ALMT1), Gene network

## Abstract

**Background:**

Acidity is a major contributor to fruit quality. Several organic acids are present in apple fruit, but malic acid is predominant and determines fruit acidity. The trait is largely controlled by the *Malic acid* (*Ma*) locus, underpinning which *Ma1* that putatively encodes a vacuolar aluminum-activated malate transporter1 (ALMT1)-like protein is a strong candidate gene. We hypothesize that fruit acidity is governed by a gene network in which *Ma1* is key member. The goal of this study is to identify the gene network and the potential mechanisms through which the network operates.

**Results:**

Guided by *Ma1*, we analyzed the transcriptomes of mature fruit of contrasting acidity from six apple accessions of genotype *Ma*_ (*MaMa* or *Mama*) and four of *mama* using RNA-seq and identified 1301 fruit acidity associated genes, among which 18 were most significant acidity genes (MSAGs). Network inferring using weighted gene co-expression network analysis (WGCNA) revealed five co-expression gene network modules of significant (*P* < 0.001) correlation with malate. Of these, the *Ma1* containing module (Turquoise) of 336 genes showed the highest correlation (0.79). We also identified 12 intramodular hub genes from each of the five modules and 18 enriched gene ontology (GO) terms and MapMan sub-bines, including two GO terms (GO:0015979 and GO:0009765) and two MapMap sub-bins (1.3.4 and 1.1.1.1) related to photosynthesis in module Turquoise. Using Lemon-Tree algorithms, we identified 12 regulator genes of probabilistic scores 35.5–81.0, including MDP0000525602 (a LLR receptor kinase), MDP0000319170 (an IQD2-like CaM binding protein) and MDP0000190273 (an EIN3-like transcription factor) of greater interest for being one of the 18 MSAGs or one of the 12 intramodular hub genes in Turquoise, and/or a regulator to the cluster containing *Ma1*.

**Conclusions:**

The most relevant finding of this study is the identification of the MSAGs, intramodular hub genes, enriched photosynthesis related processes, and regulator genes in a WGCNA module Turquoise that not only encompasses *Ma1* but also shows the highest modular correlation with acidity. Overall, this study provides important insight into the *Ma1*-mediated gene network controlling acidity in mature apple fruit of diverse genetic background.

**Electronic supplementary material:**

The online version of this article (doi:10.1186/s12864-015-1816-6) contains supplementary material, which is available to authorized users.

## Background

Apple fruit acidity refers to the sensory intensity of tartness of fruit flesh tissues. The stronger the tartness taste, the higher the fruit acidity levels. Chemically, fruit acidity can be quantified by measuring fruit juice pH and/or titratable acidity (TA). It has been shown that several organic acids are present in mature apple fruit, including malic acid, quinic acid, citric acid and others, but malic acid consists of more than 90 % of the total [1–3], thereby largely determining fruit acidity. For dessert apples, fruit acidity could be grouped into three categories by TA: low (<3.0 mg/ml), normal (3.0–10.0 mg/ml), and high (>10.0 mg/ml), and categories high and low are not acceptable [4]. This makes fruit acidity an essential quality component not only for determining the fate of existing varieties, but also for prompting routine evaluations of acidity levels in apple breeding as genotypes of fruit acidity beyond the acceptable normal range can make up 25–50 % in breeding populations. As a result, fruit acidity has long been an important subject area of investigations in apple genetics.

Apple fruit acidity is primarily controlled by the major gene or QTL on chromosome 16, called *Malic acid* (*Ma*) alongside a significant QTL on chromosome 8 and a few other QTLs with relatively smaller effects [4–9]. With regard to the allelic interactions at the major QTL *Ma*, normal/high acid allele (*Ma*) is largely dominant over the low acid allele (*ma*). Genotypes *MaMa* and *Mama* have fruit of normal to high acidity while genotype *mama* of low acidity with little or none commercial value. To focus on the most important genetic factor, we [10] and others [11] have recently isolated the major QTL *Ma*. The common findings were that the *Ma* locus harbors two new members of the *Aluminum*-*activated Malate Transporter1* (*ALMT1*) gene family, called *Ma1* and *Ma2*. The studies further found that *Ma1* was expressed in significantly positive correlation with fruit acidity levels while the expression of *Ma2* was barely detectable in both high and low acid fruit, suggesting that it was gene *Ma1* rather than *Ma2* that was the very gene underlying *Ma* [10, 11]. In addtion, a detailed analysis of the allele specific DNA sequences of *Ma1* indicated that a single base mutation that would stop the protein translation process prematurely was almost completely associated with low acidity in a diverse set of apple germplasm studied, suggesting that the low acidity is caused by the malfunction of the MA1 protein due to the deduced truncation at the C-terminus [10].

These latest findings have markedly increased our understanding on fruit acidity, but many questions remain to be answered. For example, how does the landscape of transcriptomes differ between genotypes *Ma*_ (*MaM*a and *Mama*) and *mama* at maturity stage? If fruit acidity is governed by a gene network in which *Ma1* is the genetic determinant, what would the other possible members in the network? What are the potential biological process and/or regulatory mechanisms responsible for the contrast acidity levels between genotype groups *Ma*_ and *mama*? The development of mRNA sequencing (RNA-seq) technology that unlocks the power of high throughput of next generation sequencing has provided an ideal means to address these questions. Since its inception [12, 13], RNA-seq has been rapidly adapted in transcriptomics studies in plants such as *Arabidopsis* [14], grape [15], maize [16] and rice [17]. In apple, RNA-seq based studies have recently been reported as well [18–24]. More importantly, to begin resolving the low coverage issue of the current version of apple reference transcriptome, we have improved it with RNA-seq reads from fruit of Golden Delicious (GD), the source of the reference genome [25], which is available at the Genome Database for Rosaceae (GDR) [26]. We have also used the improved reference transcriptome through RNA-seq approach to construct a co-expression gene network associated with developmental regulation of malate levels varying from 5.2 to 14.5 mg/g fw (normal to high acidity) in developing fruit of ‘Golden Delicious’ of genotype *Mama* [24].

The objectives of this study are to address the questions aforementioned. To do so, we first sequenced 30 RNA-seq libraries representing transcriptomes of mature fruit from ten apple varieties of genotypes *MaMa*, and *Mama* and *mama*, and then mapped the RNA-seq reads against the improved apple reference transcriptome [25]. Using *Ma1* as a guide gene, a series of downstream analyses was conducted, leading to identification of weighted co-expression gene network modules significantly correlated with malate, most significant acidity genes, intramodular hub genes, regulator genes, enriched gene ontology (GO) terms and MapMan sub-bins, and others. To the best of our knowledge, this is first report attempting to understand the *Ma1*-mediated gene network regulating fruit acidity in apples of diverse genetic background.

## Methods

### Plant materials and fruit acidity quantification

Ten apple varieties of known genotype at the *Ma* locus were chosen, including four of *mama*–Britegold (B), Sweet Delicious (S), Novosibirski Sweet (N) and PI323617 (P), four of *Mama*–Fuji (F, Red Sport Type 2), Rome Beauty Law (R), Cox’s Orange Pippin (C) and Jonathan (J), and two of *MaMa*–Empire (E) and Granny Smith (G). Genotypes *Mama* and *MaMa* were jointly designated *Ma*_ to represent a group that had at least one functional allele of *Ma1*. The trees were budded onto rootstock P22 and grown in a research orchard of Cornell University at Geneva, New York. Fruit of three replicates per variety and 8–10 fruit per replicate were harvested at maturity from 2 to 3 trees in fall 2012 (Additional file 1: Table S1). Each fruit was cross-sectioned into two halves: one half were used for maturity evaluation and fruit juice extraction, and the other half were sliced and immediately frozen in liquid nitrogen for RNA isolation and for quantitation of malate and other metabolites. The evaluation of fruit maturity and fruit acidity was conducted as previously described [4]. Fruit of Cornell Starch Index 4.0–6.0 [27] were considered matured and only matured fruit (with the core removed) were used for analysis. Two indicators of fruit acidity were measured: pH by a pH meter (Accumet AB15, Fisher Scientific, PA, USA) and titratable acidity (TA, malic acid equivalent) by an autotitrator (Metrohm 848 Titrino Plus with 869 compact sample changer, Metrohm, Herisau, Switzerland).

Fruit organic acids were extracted and measured using an Agilent 7890A GC/5795C MS (Agilent Technology, Palo Alto, CA, USA) with the same configurations and settings as described previously [24]. Metabolites were identified by comparing fragmentation patterns with those in mass spectral libraries and quantified based on standard curves for each metabolite and the internal standard ribitol.

### RNA-seq library construction and data analysis

Fruit RNA isolation and RNA-seq library construction were performed as previously described [25]. Briefly, total RNA was isolated from 3 g of ground fruit flesh tissues and then treated with DNase I (amplification grade, Invitrogen/Life Technologies, Carlsbad, CA). For mRNA isolation and strand specific RNA-seq library construction, NEBNext Poly(A) mRNA Magnetic Isolation Module and NEBNext Ultra Directional RNA Library Prep Kit for Illumina (New England Biolabs, Ipswich, MA) were used. The mRNA was isolated from 5 μg of total RNA and was fragmented at 94 °C for 10 min. The first strand cDNA was reverse transcribed from the fragmented mRNA with dATP mix and second strand cDNA was synthesized from the first strand cDNA with dUTP mix. The resulting double strand cDNA was end-repaired, adaptor-ligated and size-selected, and then followed by the USER enzyme digestion of the second strand cDNA. The intact modified first strand cDNA was PCR-enriched for 14–16 cycles to obtain the individual sequencing ready RNA-seq libraries. These libraries were multiplexed in equal amount for single end 100-base sequencing in three lanes of HiSeq 2000 (Illumina, San Diego, CA) at the Cornell University Biotechnology Resource Center (Ithaca, NY). The ten apple varieties were sequenced in three biological replicates with one replicate per lane.

Illumina sequencing of the pooled RNA-seq libraries generated 30 FASTQ files of sequences with a total of 615.6 million raw reads (Additional file 2: Table S2). The raw reads were fed into Bowtie [28] to remove bar codes and adapters and then aligned to the rRNA reference sequences allowing up to three mismatches, which were downloaded from the SILVA rRNA database (http://www.arb-silva.de) [29]. The rRNA depleted reads were imported into CLC Genomics Workbench v6.5 (CLCBio, Cambridge, Massachusetts) to trim low quality reads and/or bases using the quality limit of 0.05 and the ambiguous limit of 1. The resultant clean and high quality reads were mapped by CLC Genomics Workbench (using the minimum similarity fraction of 0.98, the minimum length fraction of 0.8 and the maximum number of hits of 10) against the improved apple reference transcriptome [25], which is available at URL http://www.rosaceae.org/species/malus/malus_x_domestica/CU_RNA_seq_genes of GDR [26]. In addition to 53,654 of the 63,541 originally predicted genes or MDPs (the three letters prefixing gene IDs in apple), the reference transcriptome includes 17,524 novel transcripts [25]. For convenience, these novel transcripts will be referred to ‘genes’ and named ‘G######s’ as in ‘G101234’, and ‘MDP0000’ (e.g. MDP0000252114) in the original gene IDs will be abbreviated to ‘M’ (e.g. M252114) hereafter. Gene expression levels were calculated and represented by reads per kilobase of exon model per million mapped reads (RPKM) [12]. Genes of RPKM >0.3 were considered expressed according to a previous study [30].

### Identification of fruit acidity associated genes (FAAGs)

Fruit acidity associated genes (FAAGs) were defined for those that were differentially expressed between genotype groups *Ma*_ and *mama* and/or those that were expressed similarly as the guide gene *Ma1*. To identify differentially expressed genes (DEGs) between the two genotype groups, the gene expression data RPKMs were subjected to Baggerly’s test [31]. To control the false discovery rate (FDR), the original P values in Baggerly’s test were adjusted for multiple testing using Benjamini-Hochberg correction [32]. The cutoff for declaring a gene expressed differentially was P_FDR_ <0.05. However, any genes with an RPKM range <5 in the 29 RNA seq samples were removed from the list. To identify genes expressed similarly as *Ma1*, two filters were applied: 1) the mean difference in RPKM between the two genotype groups was greater than ten (8.53 for *Ma1*), and 2) the fold change in mean RPKM between the two genotype groups was greater than 1.5 (1.69 for *Ma1*). Genes passed both filters were considered of similar expression as *Ma1*.

### Inferring fruit acidity associated co-expression gene network modules

The co-expression gene network modules (highly co-expressed clusters of genes) were inferred from the fruit acidity associated genes (FAAGs) using weighted gene co-expression network analysis (WGCNA), an R package [33]. Before network inferring, the expression data (RPKM) were normalized by square root transformation. The automatic one-step network construction and module detection method with default settings were used, which include an unsigned type of topological overlap matrix (TOM), a power β of 6, a minimal module size of 30, and a branch merge cut height of 0.25. The module eigengene (the first principal component of a given module) value was calculated and used to test the association of modules with acidity in the ten genotypes of 29 samples. Gene significance (GS, the correlation between gene expression and acidity), total network connectivity (kTotal), and module membership (MM) that is also known as eigengene-based intramodular connectivity (kME), were calculated for each of the 1301 FAAGs in the ten modules. The most significant module (Turquoise) was visualized using Cytoscape 3.1 [34] and were also analyzed using Network Analyzer [35], a Cytoscape plugin.

### Identification of regulator genes

Detection of regulator genes was performed using Lemon-Tree software [36], which is an improved and ‘one-stop shop’ version of the stochastic Bayesian module network algorithm Learning Module Networks (LeMoNe), which requires run scripts on both MATLAB and Perl [37]. Using expression data from the 1301 FAAGs, ten independent Gibbs sampler runs [38] were conducted with Lemon-Tree. From these ten runs, we were able to generate a single set of tight independent clusters, and then select 50 (Clusters 0–49) of at least ten genes per cluster for testing 96 candidate regulators. The candidate regulators were genes annotated as transcription factors or signal transducers in GO terms and/or MapMan (sub-) bins in the 1301 genes. We considered candidate regulators as regulators when their probabilistic (*P*.) scores associated with their assignment to any of the 50 tight clusters were within top 1 %.

### Functional annotation and enrichment analysis of WGCNA modules

We previously used both MapMan bins [39] and Gene Ontology (GO) terms to annotate the improved apple reference transcriptome [24, 25]. Briefly, for annotation with MapMan (sub-) bins, several databases were BLAST-searched with the apple reference transcriptome of 71,178 genes using Mercator, a web-based search tool [40]. The searched databases include: TAIR-Arabidopsis TAIR proteins (release 10), PPAP-SwissProt/UniProt Plant Proteins, CHLAMY-JGI Chlamy release 4 Augustus models, ORYZA-TIGR5 rice proteins, KOG-Clusters of orthologous eucaryotic genes database, CDD- conserved domain database, and IPR-interpro scan. For GO term annotations, relevant information for apple genes (MDPs) is available at GDR [26] and was directly adapted. However, GO terms for the new transcripts [25] were obtained by BLAST2GO program [41] together with BLASTx search against the NCBI Reference Proteins database (cutoff E = 10^−6^). Enrichment analyses of the WGCNA modules were all conducted by hypergeometric tests using CLC GW against the same background of 39,679 expressed genes. The hypergeometric test is similar to the unconditional GOstats test [42], and the cutoff for significant enrichment is P_FDR_<0.05. Depending upon actual test, the background was defined by 39,679 annotated genes in MapMan (sub-) bins, by 16,153 in GO biological process (BP), or by 21,612 in GO molecular functions (MF).

### qRT-PCR analysis

qRT-PCR analyses were conducted with the same total RNA samples (after DNase I treatment) used for the RNA-seq library construction. Two micrograms of total RNA were reverse transcribed using the Superscript III RT module (Invitrogen/Life technology, Carlsbad, CA). The resulting first strand cDNA was diluted by 5-fold and used as templates for qRT-PCR analysis on a LightCycler 480 (Roche, Indianapolis, IN), where an apple actin gene (EB136338) was served as reference. The primer sequences for the reference gene and the eight target genes were listed (Additional file 3: Table S3). The actual qRT-PCR reactions and subsequent expression analysis were conducted similarly as described previously [24].

### Statistical analyses

Statistical analyses, such as ANOVA, Student’s *t* test and Tukey’s HSD (honest significant difference) test, were performed using JMP Pro10 (SAS, Cary, NC).

## Results

### Fruit metabolite profiling and acidity evaluation

**Fig. 1:**
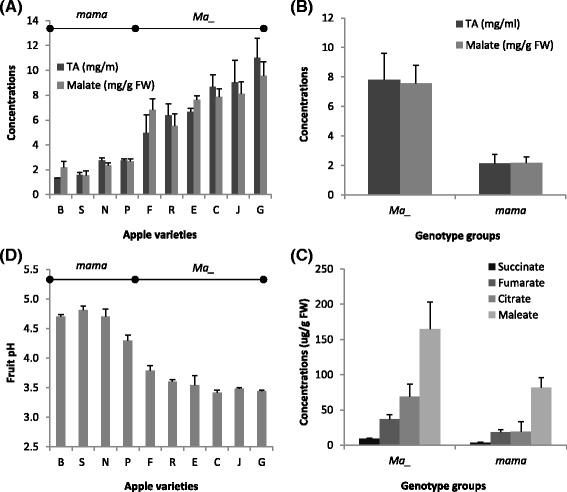
Evaluation of fruit acidity and causal organic acids. Standard deviations were shown with the *error bars*. **a** TA and malate concentrations measured in the ten apple varieties, which were abbreviated as the following: *B* Britegold, *N* Novosibirski Sweet, *P* PI323617, *S* Sweet Delicious,* C* Cox’s Orange Pippin, *E* Empire, *F* Fuji, *G* Granny Smith, *J* Jonathan, *R* Rome Beauty Law. The first four are of genotype *mama* whereas the last six of genotype *Ma*_. **b** Mean TA and malate concentrations in genotype groups *Ma*_ and *mama*. **c** Concentrations of succinate, fumarate, citrate and maleate in genotype groups *Ma*_ and *mama*. **d** Fruit pH readings in the ten apples

Fruit metabolite profiling was conducted using GC-MS with three biological replicates in the ten apple varieties. A total of 19 metabolites were quantified, including 12 soluble sugars and seven organic acids. Among the 12 sugars, only sorbitol showed a significantly (*P* = 0.026, ANOVA) higher concentration in genotype group *mama* (7.4 ± 2.0 mg/g FW) than in *Ma*_ (5.4 ± 1.9 mg/g FW). Other sugars, especially the highly abundant fructose, sucrose and glucose, had similar levels across these varieties. Among the seven organic acids (malate, dehydroascorbate, maleate, succinate, fumarate, citrate and quinate), malate was the most abundant (87.2 ± 7.2 % of the total acidity) while quinate (8.3 ± 6.7 %) and maleate (2.5 ± 0.8 %) were distantly followed as the second and third abundant, respectively. Malate concentrations varied significantly (*P* = 1.03E-9) in the ten apple varieties (Fig. 1a), and were more than 3-fold higher in genotype group *Ma*_ (7.58 ± 1.23 mg/g FW) than in *mama* (2.16 ± 0.41 mg/g FW) (Fig. 1b). The concentrations of maleate, succinate, fumarate and citrate were also significantly higher in genotype group *Ma*_ than in *mama* (*P* < 0.0001), but they were at much lower levels (at μg/g FW) (Fig. 1c). However, quinate showed no significant difference between groups *Ma*_ and *mama*. The trend of fruit juice titratable acidity (TA) was similar to that of malate in the ten varieties (Fig. 1a, b) while an expected reverse trend was observed for fruit juice pH (Fig. 1d).

### Transcriptome analysis in genotype groups *Ma*_ and *mama*

RNA-seq data analysis was performed using the improved apple reference transcriptome [25]. After removing reads derived from rRNA and/or of low quality, the reads for RNA-seq mapping were 462.9 million in total (Additional file 2: Table S2). Overall the total mapped and uniquely mapped reads were 331.6 million (70.5 %) and 281.4 million (59.8 %), respectively. The mean mapped reads per sample were 11.1 ± 4.1 million in total and 9.4 ± 3.5 million in unique. However, the sample for replicate I of Granny Smith was excluded from the downstream analysis as 75.0 % of its raw reads was removed by the rRNA and quality filters (Additional file 2: Table S2).

**Fig. 2:**
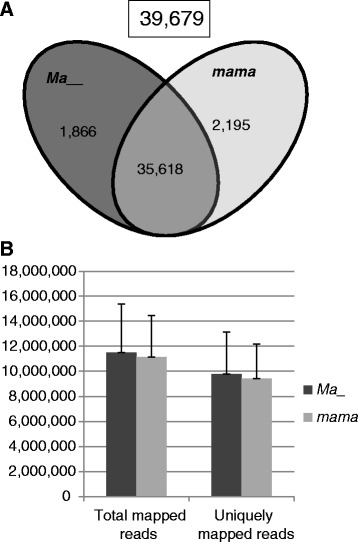
Summary of RNA-seq data analysis. **a** Venn diagram representation of the number of genes expressed (39,679) in genotype groups *Ma*_ (*dark grey*) and *mama* (*light grey*). The cutoff is group mean RPKM >0.3. **b** Mean number of total and uniquely mapped reads per sample in genotype groups *Ma*_ and *mama*. Standard deviations were shown with the *error bars*

The maximum number of expressed genes was 52,102 in this study, a tally counting all genes of RPKM >0.3 in at least one of the 29 samples. However, measuring by the threshold in group means (RPKM >0.3 in at least one of the two group means), the expressed genes were 39,679 (Fig. 2a). Of these, 35,618 were expressed in both genotype groups, and 1866 and 2195 were specifically in *Ma*_ and *mama*, respectively (Fig. 2a).

The total mapped reads of the expressed genes were 11.5 ± 3.9 million per sample in the *Ma*_ group and 11.1 ± 3.3 million per sample in *mama* (Fig. 2b). The uniquely mapped reads per sample in the *Ma*_ and *mama* groups were 9.8 ± 3.3 million and 9.4 ± 2.8 million, respectively (Fig. 2b).

### Identification of malate associated co-expression gene network modules using WGCNA

**Fig. 3:**
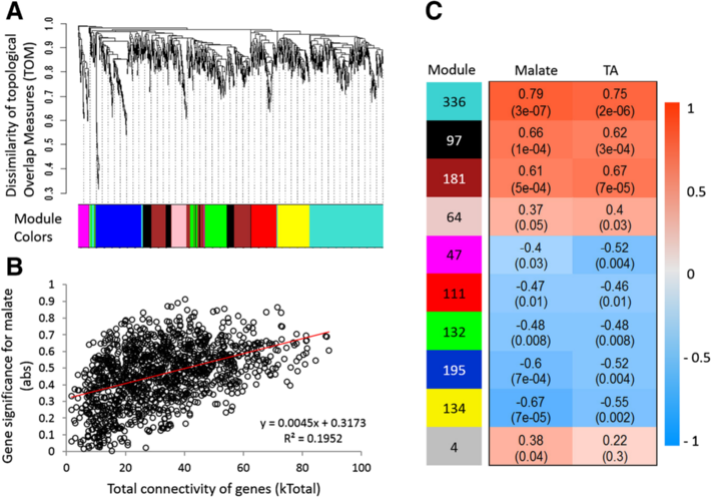
Weighted gene co-expression network analysis (WGCNA) of fruit acidity associated genes (FAAGs) in the *Ma*_ and *mama* genotype groups. **a** Hierarchical cluster tree showing ten modules of co-expressed genes. Each of the 1301 FAAGs is represented by a leaf in the tree while each of the ten modules by a major tree branch. The *lower panel* shows modules in designated colors. **b** Correlation between gene total network connectivity and absolute gene significance for malate in the whole co-expression gene network. **c** Module-fruit acidity correlations and corresponding *p*-values (*in parenthesis*). The *left panel* shows ten modules (*Turquoise*, *Black*, *Brown*, *Pink*, *Magnate*, *Red*, *Green*, *Blue*, *Yellow* and *Grey*) and the number of their member genes. The *right panel* is a color scale for module trait correlation from −1 to 1

Transcriptome-wide comparison of the 39,679 expressed genes revealed 1301 fruit acidity associated genes (FAAGs), including 633 (including *Ma1*) of differentially (P_FDR_ <0.05) expressed genes (DEGs) between groups *Ma*_ and *mama*, and 668 expressed similarly as the guide gene *Ma1* (Additional file 4: Table S4). Using WGCNA, a co-expression gene network was constructed from the 1301 FAAGs (Fig. 3a). Within the network, the total network connectivity of genes showed a significant correlation (r^2^ = 0.1952, *p* = 4.02E-12) with the gene significance for malate (Fig. 3b), suggesting that more connected genes would have higher gene significance for acidity in the 1301 FAAGs.

**Fig. 4:**
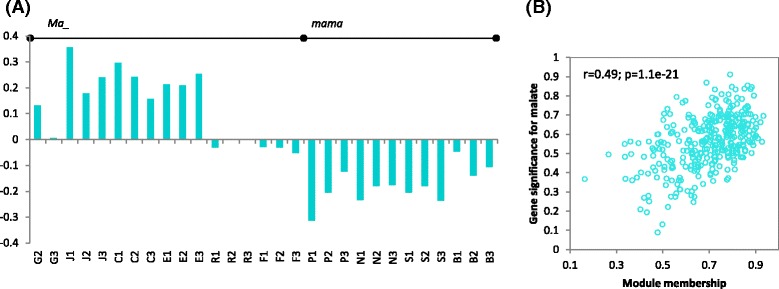
Analysis of module Turquoise. **a** Module eigengene values across the 29 samples, including 17 in *Ma*_ on *left* and 12 in *mama* on *right*. Samples are represented by the combination of a *letter* (abbreviated cultivar name, see Fig. 1’s legend) and a *number* (replicate 1, 2 or 3). **b** Correlation between module membership (MM) and gene significance (GS) for malate

Further WGCNA analysis identified ten network modules in the co-expression network, designated Turquoise, Black, Brown, Pink, Magenta, Red, Green, Blue, Yellow, and Grey (for four unassigned genes) (Fig. 3a, c). Investigating the relationships between module eigengene and acidity uncovered that the correlation coefficients varied widely from −0.67 to 0.79 in malate and from −0.55 to 0.75 in TA (Fig. 3c). The eigengenes (Fig. 4a, Additional file 5: Figure S1A) of five modules Turquoise, Black, Brown, Blue and Yellow showed significant correlations (*p* < 0.001) with malate, suggesting these five modules had greater relevance in fruit acidity although Blue and Yellow were negatively correlated (Fig. 3c). The five modules comprised 943 genes with module Turquoise being the largest of 336 genes. Notably, the guide gene *Ma1* was assigned to Turquoise, which also had the highest modular correlation (*r* = 0.75–0.79) with acidity (Fig. 3c). Inspecting the correlation between the module memberships (MM) and gene significance (GS) revealed that all modules were significant except Blue (Fig. 4b, Additional file 5: Figure S1B). Again, module Turquoise had the most significant correlation between MM and GS (*r* =0.49, *p* = 1.1E-21), indicating genes of higher MM values were more likely of greater GS in Turquoise than in others.

### Identification of the most significant acidity genes (MSAGs)

**Fig. 5:**
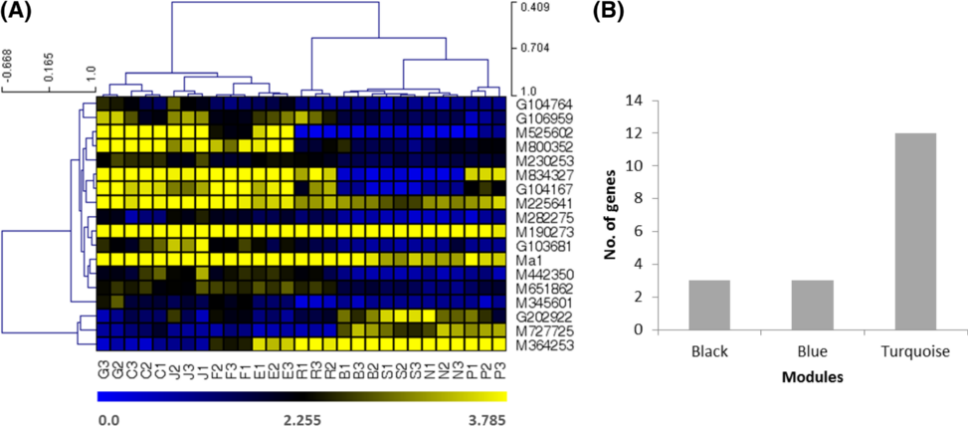
The most significant acidity genes (MSAGs). **a** Expression profile (square root of RPKM) of the 18 MSAGs. Each *row* represents a gene as listed on *right*. Each *column* stands for a sample as specified by sample names (see the legend in Fig. 1 for keys) at *lower panel*. Clustering of genes and samples was shown with distance on *left* and *top panels*, respectively. The expression of genes is color coded from low (*dark blue*) through mean (*black*) and through high (*bright yellow*). The *bright yellow color* across the samples for *Ma1* and M190293 indicate the expression of these two genes were all close to or exceeded the high end of the scale at *bottom*. **b** Distribution of the 18 MSAGs in modules. Modules with zero genes are not shown. Note that genes of IDs beginning with letter ‘G’ are referred to the novel transcripts [25], and the apple reference gene IDs are abbreviated (e.g. M250124 stands for MDP0000250124)

The 1301 FAAGs had a range of absolute gene significance (GS) for malate from 0.004 to 0.911 (Fig. 3b). We define that the most significant acidity genes (MSAGs) are those of GS for malate ≥0.801, the observed GS of the guide gene *Ma1*. This allowed to identify 18 MSAGs (Fig. 5a, Additional file 6: Table S5), including three transporters (M282275, M834327 and *Ma1*), two receptor kinases (M525602, M651862), one Ethylene-Insensitive3 (EIN3)-like transcription factor (M190273), one pyruvatedehydrogenase complex component E2 (M727725), one endo-1,4-beta-xylanase/ hydrolase (M225641), one glutamine synthetase cytosolic isozyme (G202922), one photosystem II subunit R (M800352), and seven (M364253, M442350, M345601, G103681, M230253, G106959, G104167 and G104764) of unknown function. The 18 MSAGs were distributed in three modules with 12 in Turquoise, three in Black and another three in Blue (Fig. 5b, Additional file 6: Table S5), further indicating module Turquoise is of greater relevance in acidity.

### Identification of intramodular hub genes and evaluation of their total network connectivity changes in genotype groups *Ma*_ and *mama*

**Fig. 6:**
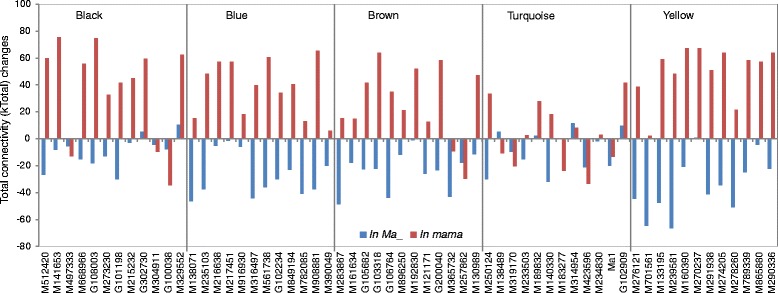
Intramodular hub genes and their total network connectivity changes in genotype groups *Ma*_ and *mama* when compared with the combined groups of both genotypes. Shown are modules *Black*, *Blue*, *Brown*, *Turquoise* and *Yellow*

To identify intramodular hub genes, we similarly used the guide gene *Ma1*, which had an MM of 0.894, ranking 11th in module Turquoise. Based on this information, we defined top 12 genes in MM values from each of the five modules as intramodular hub genes, leading to identification of 60 hub genes (Fig. 6, Additional file 7: Table S6). To evaluate how the 60 hub genes behaved in genotype groups *Ma*_ and in *mama*, their respective total network connectivity (kTotal) were calculated and compared with the overall group of both genotypes (Fig. 6, Additional file 7: Table S6). Most of the 60 hub genes showed a large increase in kTotal in *mama* while a marked reduction in *Ma*_, suggesting a major pattern of negative correlation between the hub genes’ kTotal changes and acidity levels (Fig. 6). This pattern was true in modules Blue and Yellow (both negatively correlated with acidity), largely true in Brown and Black (both positively correlated with acidity), but largely untrue in Turquoise (most positively correlated with acidity), seemingly providing another means depicting the module-acidity relationship shown early (Fig. 3c).

There were three additional patterns in kTotal changes (Fig. 6): The first was observed only on M138489 in Turquoise, i.e. a reverse trend by having an increased kTotal in *Ma*_ and a decreased kTotal in *mama*. M138489 encodes a leucine-rich repeat receptor kinase (Fig. 6). The second was represented by *Ma1*, i.e. the kTotal was decreased in both *Ma*_ and *mama*, suggesting reduced kTotal of *Ma1* in *mama* might have important ramifications for low acidity. This pattern was also observed on M319170 (encoding an AtIQD2 like calmodulin (CaM) binding protein involved in calcium signaling), M423596 (a homeobox transcription factor) and M183277 (a hypothetic protein) in Turquoise, M497333, M304911 and G100038 in Black, and M365732 and M257862 in Brown (Fig. 6, Additional file 7: Table S6). The third pattern was characterized by an increased kTotal in both *Ma*_ and *mama*, which was observed on M189832 (a metalloendopeptidase in protein degradation), M314954 (a WD-40 repeat family protein) and G102909 (a hypothetical protein) in Turquoise, and G302730 and M329552 in Black (Fig. 6, Additional file 7: Table S6).

**Fig. 7:**
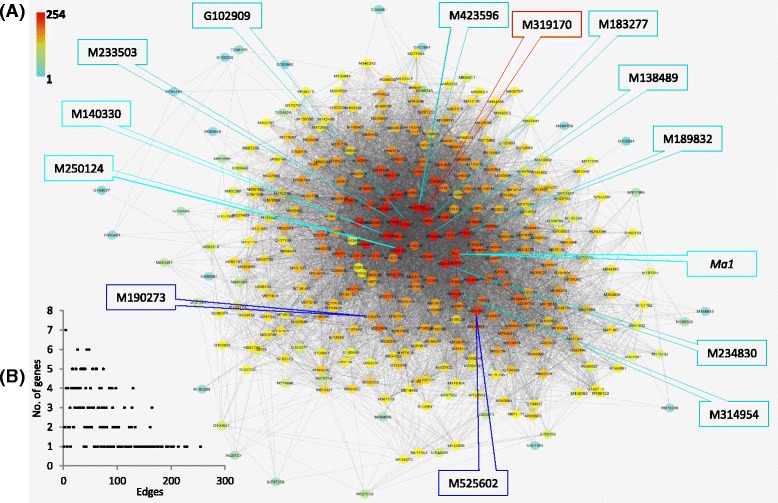
Graphic view of module Turquoise. **a** Cytoscape representation of co-expressed genes with edge weight ≥0.10. The edge number of the genes ranges from 1 (G102044) to 254 (M250124), which is color coded by the scale at *top left* from *blue* through *red*. Some important genes are noted, including 11 intramodular hub genes M250124, M138489, M233503, M189832, M140330, M183277, M314954, M423596, M234830, G102909, and *Ma1* (in *turquoise box*), two regulator genes M190273 and M525602 (in *blue box*) and one hub and regulator gene M319170 (in *red box*). **b** Distributions of edges. The 12 hub genes are among those of most edges (164–254) of weight ≥0.10

To graphically view the intramodular connectivity of these hub genes, the 12 hub genes along with other co-expressed (WGCNA edge weight >0.10) genes in module Turquoise was presented using Cytoscape (Fig. 7a). It showed that the 12 hub genes were of the most edges from 164 to 254 (Fig. 7b) and were in the module network core (Fig. 7a).

### Identification of regulator genes

**Fig. 8:**
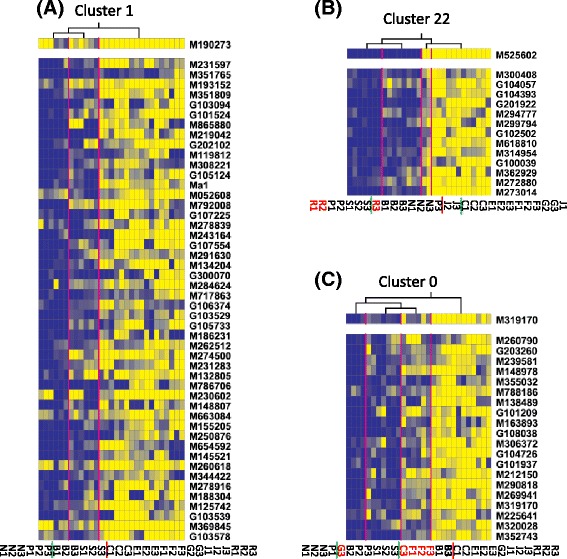
Regulator genes and their assigned tight clusters. **a** Regulator M190273 (*upper panel*) and Cluster 1 (*mid panel*) of 48 genes. The expression of genes is color coded from low (*dark blue*) through high (*bright yellow*). Each *row* stands for a gene (listed on the *right*) and each *column* for a sample named at the *bottom* (see the legend in Fig. 1 for keys). The regulator is assigned based on the hierarchical tree on *top*, which indicates how samples were clustered together with the *red vertical lines*. The *short red vertical line* in the sample list shows where the two primary clades diverge while the *short green vertical line* marks where the secondary clades depart within a primary clade. The genotype names in *red* indicate they are not in agreement with one of the two primary clusters *Ma*_ or *mama*. **b** Regulator M525602 and Cluster 22 of 13 genes. **c** Regulator M319170 and Cluster 0 of 20 genes. Elements in (**b**) and (**c**) and their contents, formats and messages are the same as those in (**a**)

Regulator genes are not readily identifiable from the WGCNA co-expression network modules as they are non-directed. To identify regulator genes, we used Lemon-Tree software [36] first to generated 50 tight clusters (Clusters 0–49) covering 839 of the 1301 FAAGs through ten Gibbs sampler runs [38], and then to examine the 96 candidate regulators (transcription factors or signal transducers annotated in the 1301 FAAGs) with the clusters. To statistically validate the results obtained from the 96 candidate regulators, another 96 randomly selected genes were used as control (CK) and examined in parallel with the 96 candidate regulators. As expected, the probabilistic (*P*.) scores for genes assigned as a regulator for the 50 tight clusters showed significant difference (*p* = 0 in z test) between the 96 candidate regulators (0.1–81.0) and CK (0.1–6.3). At the top 1 % of *P*. scores (35.5–81.0), 12 of the 96 candidate regulators were identified as regulators and assigned to 21 of 50 tight clusters (Additional file 8: Table S7). Of the 12 regulators, five (M190273, M525602, M319170, M239684 and M134341) were from module Turquoise (Fig. 8, Additional file 9: Figure S2, Additional file 10: Figure S3), two (M753318 and M175481) from Brown (Additional file 10: Figure S3), and five from modules Green, Red and Pink which were irrelevant for acidity (Additional file 8: Table S7). Below are brief descriptions of three regulator genes, which were among the 18 MSAGs or among the 12 most connected intramodular hub genes in module Turquoise.

The first is M190273, encoding an EIN3-like transcription factor. M190273 was assigned to six tight clusters 1, 8, 9, 21, 40 and 45 with *P*. scores 40.7–81.0 (Fig. 8a, Additional file 8: Table S7, Additional file 9: Figure S2). Notably, Lemon-Tree clustered *Ma1* into Cluster 1, to which M190273 was assigned as a regulator with the highest *P*. score (81.0) (Fig. 8a), suggesting M190273 might potential regulate *Ma1*. M190273 was one of the 18 MSAGs (Fig. 5a, Additional file 6: Table S5) and of high MM value (0.866) in module Turquoise. In the six M190273 regulated clusters, the fruit samples were all clustered into two primary clades, which were nearly perfectly alongside the line dividing the two genotypes *Ma*_ and *mama* (Fig. 8a, Additional file 9: Figure S2). Such clustering pattern of fruit samples were not observed in any of the remainder 15 clusters regulated by the other 11 regulator genes (Fig. 8, Additional files 9, 10: Figures S2, 3), further supporting the potential regulatory role of M190273 in fruit acidity. The second is M525602, encoding a receptor-like protein kinase. M525602 was assigned to cluster 22 with *P*. score 37.8 (Fig. 8b). Similar to M190273, M525602 is also one of the 18 MSAGs (Fig. 5a) that had a high MM value (0.884) in module Turquoise. The third is M319170, one of the 12 intramodular hub genes in module Turquoise (Fig. 6). M319170 was assigned to cluster 0 with *P*. score 36.7 (Fig. 8c). As aforementioned, M319170 encodes an AtIQD2-like CaM binding protein involved in calcium signaling and behaved similarly as *Ma1* in kTotal changes in genotype groups *Ma*_ and *mama*.

### Detection of enriched GO terms and MapMan (sub-) bins in network modules

To detect enriched GO terms and MapMan (sub-) bins in the five significant modules, a series of hypergeometric tests were performed against the background of 39,679 expressed genes. Overall, eight GO terms and ten MapMan sub-bins were enriched (P_FDR_ <0.05) in modules Turquoise, Brown, Blue and Yellow, but not in Black, and no terms were repressed (Table 1, Additional file 11: Table S8). Due to multifaceted roles of genes and due to both GO and MapMan systems were used, some of the enriched terms had the same or mostly overlapping member genes. In total, the 18 enriched terms covered 77 non-redundant genes, including 17 in module Turquoise, 9 in Brown, 46 in Blue and 5 in yellow (Table 1, Additional file 11: Table S8).

**Table 1:**
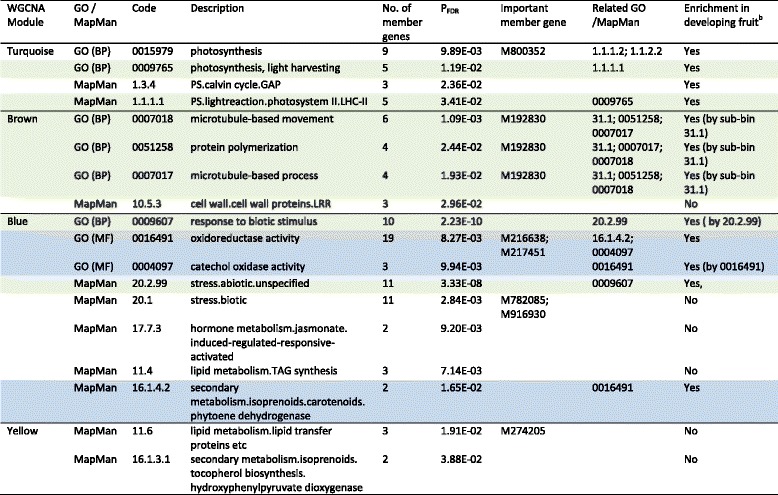
GO terms and/or MapMan (sub-) bines significantly enriched in WGCNA modules significantly correlated with fruit acidity^a^

^a^The GO terms and/or MapMan (sub-) bins that have the same or mostly overlapping member genes in the same module are highlighted with same color(s)

^b^Bai et al. [24]

In Turquoise, two GO (BP) terms (GO:0015979 and GO:0009765) and two MapMan sub-bins (1.3.4 and 1.1.1.1) were enriched, which are all related to photosynthesis (Table 1, Additional file 11: Table S8). However, GO:0009765 and sub-bin 1.1.1.1 had the same five member genes. Notably, M800352, a member in GO:0015979, is one of the 18 MSAGs (Fig. 5a, Table 1, Additional file 11: Table S8).

In Brown, three GO (BP) terms and one MapMan sub-bin 10.5.3 (cell wall leucine-rich repeat family protein) were enriched (Table 1, Additional file 11: Table S8). The three GO terms were highly related as the four member genes in GO:0051258 (protein polymerization) and GO:0007017 (microtubule-based process) were identical and were also among the six member genes in GO:0007018 (microtubule-based movement). M192830, a common member gene in these GO terms, is one of the 12 hub genes in Brown (Fig. 6).

In Blue, we detected three GO terms and five MapMan sub-bins, the most in this study (Table 1, Additional file 11: Table S8). But the ten member genes in GO:0009607 (response to biotic stimulus) were fully covered by the 11 genes in sub-bin 20.2.99 (abiotic stress unspecified). Similarly, the two genes in sub-bin 16.1.4.2 (carotenoid phytoene dehydrogenase) and three genes in GO:0004097 (catechol oxidase activity) were covered by the 19 genes in GO: 0016491 (oxidoreductase activity). Sub-bins 11.4 (triacylglycerol synthesis), 17.7.3 (jasmonate responsive) and 20.1 (biotic stress) were unique. Remarkably, four of the 12 hub genes in Blue were present, i.e. M216638 and M217451 in GO: 0016491, and M782085 and M916930 in sub-bin 20.1 (Fig. 6, Table 1, Additional file 11: Table S8).

In Yellow, two MapMan sub-bins 11.6 (lipid transfer proteins) and 16.1.3.1 (hydroxyphenylpyruvate dioxygenase) were enriched, and M274205 from sub-bin 11.6 is one of the 12 hub genes in Yellow (Fig. 6, Table 1, Additional file 11: Table S8).

### qRT-PCR confirmation of gene expression

**Fig. 9:**
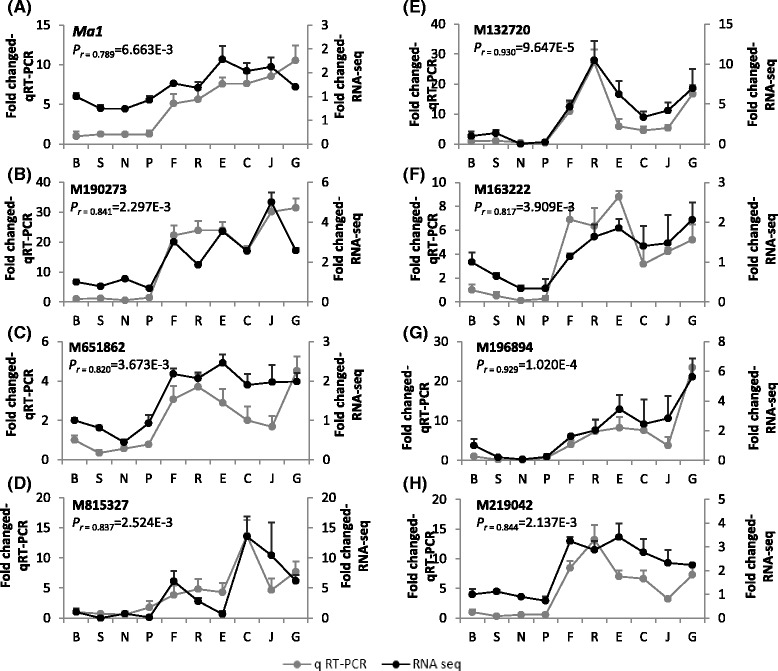
Expression confirmation of eight selected genes using qRT-PCR. **a**-**h** The normalized expression of target genes relative to a control gene (actin) in qRT-PCR was shown in *light grey*, and their corresponding RPKM values from RNA-seq were in *black*. The correlation coefficient (*r*) and associated *p* value (*n* = 10) were shown accordingly. Standard deviations were shown with the *error bars*

To evaluate if and how the RPKM values reflected the gene expression levels, a set of eight genes were analyzed using qRT-PCR (Fig. 9). The eight genes include *Ma1*, the EIN3-like regulator M190273, another most significant gene for acidity M651862 encoding a protein serine/threonine kinase, and five others. The data not only confirmed that the relative expressions of the eight genes in qRT-PCR were significantly (*p* = 6.663E-3 to 9.647E-5) correlated with their RPKM values in RNA-seq, but also confirmed their differential expression between the two genotypes groups *Ma*_ and *mama*.

## Discussion

### Identification of individual fruit acidity associated genes

In this study, ten apple varieties were carefully chosen to represent the two genotype groups *Ma*_ (*MaMa* and *Mama*) and *mama* of contrast fruit acidity levels (normal/high vs low). These varieties were grafted on the same rootstock P22 and grown in the same orchard with the same management routines to curtail the environmental impacts on gene expression. However, since the apples are of diverse genetic background and matured at varying dates, fruit transcriptome at maturity were inevitably influenced by these genetic and environmental variables. To minimize such biases, three replicates per variety and at least four varieties per genotype group were sampled and used for RNA-seq analysis. This strategy appeared to be appropriate and adequate for the defined objectives in this study as we identified 1301 fruit acidity associated genes (FAAGs). In developing fruit of ‘Golden Delicious (GD)’ of varying malate levels, 3066 differentially expressed genes (DEGs) were identified [24]. In comparison of the two lists of genes, 1050 (80.7 %) of the 1301 FAAGs were not present in the 3066 DEGs, suggesting most genes relevant for fruit acidity variation across diverse genetic background were largely different from those important for malate variations in the developing fruit of GD. Overall, we found 37,484 genes expressed (RPKM >0.3) in the *Ma*_ group and 37,813 genes in the *mama* group. As there were 35,618 genes expressed in both groups, the total number of expressed genes was 39,679 (Fig. 2a). Varying RPKM thresholds were employed in published studies [43, 44], we view RPKM >0.3 used previously [30] fit this set of RNA-seq data well. Of the 39,679 expressed genes, 1301 were regarded FAAGs. Given the significant correlation between RPKM in RNA-seq and relative expression in qRT-PCR in the eight genes tested, we consider that the majority, if not all, of the genes relevant for fruit acidity variation at maturity in this study were included in the 1301 genes.

### Co-expression gene network modules and GO terms and MapMan (sub-) bins relevant for fruit acidity

WGCNA [33, 45] is one of the programs used widely for inferring co-expression network modules [46–49]. Using WGCNA, we identified five modules highly associated with fruit acidity (Fig. 3c). Hypergeometric tests identified eight GO terms and ten MapMan (sub-) bins that were enriched in four of the five modules (Table 1, Additional file 11: Table S8).

In the most significant module Turquoise that includes *Ma1*, the two enriched GO terms and two MapMan sub-bins are all related to photosynthesis (Table 1, Additional file 11: Table S8). In the developing fruit, the two GO terms and the two MapMan sub-bins were also enriched [24], and 11 (64.7 %) of the 17 member genes in the four enriched terms were identified in both studies (Table 1, Additional file 11: Table S8). This suggested that fruit photosynthesis could likely be a part of the mechanism for the contrast malate levels between the two genotype groups at maturity. It was proposed that the relative more active photosynthesis in young fruit may facilitate malate biosynthesis, thereby contributing to higher malate levels in young fruit than in maturing fruit [24]. However, chloroplasts are only present in the hypodermal green tissues and the inner perivascular tissues in mature apple fruit, and are essentially absent in parenchyma cells that constitute the major portion of flesh [50, 51]. How enhanced photosynthesis activities in a fraction of cells would lead to increased overall malate levels in fruit remains to be understood.

In module Brown, the enrichment of MapMan sub-bin 10.5.3 (cell wall leucine-rich repeat family protein) was unique to this study. However, the three enriched GO terms (GO:0007018, GO:0051258 and GO:0007017) were essentially a reflection of MapMan sub-bin 31.1 (cell organization) (Table 1, Additional file 11: Table S8), which was enriched in negative malate dependent manner in developing fruit [24]. In module Blue, the enrichment of MapMan sub-bins 11.4 (triacylglycerol synthesis), 17.7.3 (jasmonate responsive) and 20.1 (biotic stress) were identified in this study only. But GO:0016491 (oxidoreductase activity), and MapMan sub-bins 20.2.99 (abiotic stress unspecified) and 16.1.4.2 (carotenoid phytoene dehydrogenases) were also enriched in developing fruit [24]. In module Yellow, the enrichment of two MapMan sub-bins 11.6 (lipid transfer proteins) and 16.1.3.1 (hydroxyphenylpyruvate dioxygenases) was unique in these diverse mature fruit.

Overall, 12 enriched terms (covering 53 of the 77 genes), including four from Turquoise (17 genes), three from Brown (6 genes), and five from Blue (30 genes), were also enriched in developing fruit of ‘Golden Delicious’ [24], whereas the remainder six terms (covering 24 of the 77 genes) were enriched specifically in this study (Table 1, Additional file 11: Table S8). This suggested that common and unique processes relevant for malate variations in both the diverse mature fruit and the developing fruit of ‘Golden Delicious’ exist. However, 27 of the 30 genes in Blue and all of the six genes in Brown were not found to be relevant in developing fruit although 11 of the 17 genes related to photosynthesis in Turquoise were (Table 1, Additional file 11: Table S8), suggesting the common processes may operate but most likely with their unique gene sets.

### Transcriptional regulation of fruit acidity mediated by *Ma1*

In an ongoing survey of the USDA *Malus* repository at Geneva, New York, we identified 40 diverse apple accessions of genotype *ma1ma1* using marker CAPS_1455_ that can detect the stop codon leading SNP in *ma1* [10] and found that each of them had fruit pH >4.0 (Xu, unpublished data), a typical low fruit acidity characteristic in genotype group *mama*. These data, together with the previous studies [4, 10, 11], suggested us that *Ma1*, a member of the ALMT1 gene family [52], is the genetic determinant underlying the *Ma* locus controlling acidity levels in mature apple fruit although the functional genetic complementation data are not available.

*Ma1* is thought to exert its effect on fruit acidity by both the truncation leading mutation and expression levels, where the mutation plays greater role [10]. In this study, *Ma1* showed not only high gene significance for malate (Fig. 5a), but also high intramodular connectivity in module Turquoise (Figs. 6, 7), further supporting this notion. However, in our previous effort to understand the developmental regulation of malate levels in the developing fruit of ‘Golden Delicious’, *Ma1* was not present in the major co-expression gene network due to its less significant (*p* = 0.03) correlation with malate although the involvement of *Ma1* was clear [24]. This is likely caused by the fact that the study focused on developing fruit of a single variety (‘Golden Delicious’) of genotype *Mama* that had malate levels equivalent to the normal to high acidity range during fruit development, i.e. the truncation mutation effect of *Ma1* for low acidity in genotype group *mama* was absent.

The current models accounting for the ALMT1 mediated plant tolerance to soil aluminum toxicity in *Arabidopsis* and rice comprise several common elements [53, 54]. These elements include environmental stimuli (Al^3+^/H^+^), a receptor (unknown) on the plasma membrane that interacts with Al^3+^/H^+^ to initiate a signal transduction pathway (unknown), transcription factors (STOP1 in *Arabidopsis* and ART1 in rice, both of which are C_2_H_2_ zinc finger transcription factors, i.e. TFs), protein kinase (unknown) activating the TFs by phosphorylation, and responsive genes ALMT1, ABC transporters (ASL1, ASL3, STAR1) and others [53, 54]. In the developing fruit of GD, several groups of genes functionally similar to these elements were co-enriched or co-suppressed in a malate dependent manner, including 14 C_2_H_2_ transcriptional factors, 27 protein kinases, and 23 receptor kinases for signaling [24]. In addition, G105811, one of 14 C_2_H_2_ transcription factors, was annotated as a STOP1-like protein by Mercator [40]. These had led to a speculation that a similar transcriptional regulation model for malate in developing fruit may exist although ALMT1 targets plasma membrane [24].

In this study, some of these functionally similar components were also identified. For example, within the 18 MSAGs, we see M525602 encoding a leucine-rich repeat (LLR) transmembrane receptor protein kinase for signaling, M651862 a protein serine/threonine kinase, M282275 an ABC transporter, and of cause *Ma1* an ALMT1 like malate transporter (Fig. 5a). Interestingly, Lemon-Tree based regulator analysis identified the LLR receptor kinase encoding gene M525602 as a regulator of Cluster 22 of 13 genes (Fig. 8b). Moreover, M250124, the most intramodular connected gene in module Turquoise (Fig. 6), encodes a protein kinase. These data suggested that the ALMT1 mediated plant tolerance models somewhat be plausible for the transcriptional regulation of fruit acidity. However, this study identified only one C_2_H_2_ zinc finger TF (M302279) in the 1301 FAAGs assigned to module Red not highly correlated with acidity. There is no evidence for C_2_H_2_ zinc finger TFs being part of the transcriptional regulation of *Ma1* mediated fruit acidity.

Identification of regulator M190273 by Lemon-Tree may have suggested an emerging mechanism for fruit acidity regulatory network mediated by *Ma1*. Again, M190273 is one of the MSAGs (Fig. 5a) as well as a highly connected gene in module Turquoise (Fig. 7). Lemon-Tree assigned M190273 as a regulator to six clusters of 183 genes in total, including Cluster 1 containing *Ma1* (Fig. 8a), Cluster 40 containing G104764 and M345601 (Additional file 9: Figure S2C), and Cluster 21 containing M651862 (Additional file 9: Figure S2D), which are all among the 18 MSAGs (Fig. 5a). Although G104764 and M345601 are of unknown function, M651862 was speculated as a possible protein kinase component in the ALMT1 model aforementioned. Another relevant observation is that samples in these six clusters were all clustered alongside the line between the two genotype groups *Ma*_ and *mama*, a pattern not observed for any other regulators, suggesting M190273 is likely a critical regulator for fruit acidity. M190273 shows some sequence similarity to EIN3-like genes, which are transcription factors important in ethylene response and signal transduction in plant [55, 56]. Previous studies documented that the root growth inhibition by aluminum stress is mediated by ethylene in bean [57], *Lotus japonicus* [58], and *Arabidopsis* is [59]. A more recent study reported that aluminum-induced malate efflux from wheat roots and tobacco cells transformed with TaALMT1 was negatively regulated by ethylene although the molecular mechanisms remain unknown [60]. These reports hint that it is possible for M190273 to be a regulator of malate in apple fruit.

M319170, one of the 12 hub genes in module Turquoise (Fig. 6), is a regulator assigned to Cluster 0 containing M225641, one of the 18 MSAGs (Fig. 5a). M319170 encodes a CaM binding protein similar to AtIQD2 involved in calcium (Ca^2+^) signaling [61]. M140330, another hub gene of module Turquoise (Fig. 6), encodes a protein similar to CaM-like3 (CML3) of role in calcium signaling [62]. Among the 18 MSAGs (Fig. 5a), M834327 encodes a calcium regulated channel similar to AtCNGC 20. It has been demonstrated that AtCNGC20 is localized to tonoplast [63] as well as plasma membrane [64], and to interact with all CaM isoforms through its IQ domain [64]. In a study investigating carbohydrate metabolism in two apple genotypes that sharply differ in malate accumulation in a way similar to those between the two genotype groups *Ma*_ and *mama* found that calcium contents were significant higher in high acid fruit than in low acid fruit [65]. These observations suggested Ca^2+^ signaling might also be an important direction for better understanding the regulation of malate levels in mature apple fruit. Interestingly, Ca^2+^ signals have also been considered one of the possible signal transduction pathways for aluminum tolerance lately [54].

## Conclusions

The most relevant finding of this study is the identification of a weighted gene co-expression network analysis (WGCNA) module (Turquoise) of 336 genes that not only encompasses *Ma1* but also shows the highest modular correlation with acidity in mature fruit of diverse genetic background. Based on Lemon-Tree algorisms, MDP0000190273, which putatively encodes an EIN3-like transcriptional factor, is likely the most important regulator of *Ma1* and its mediated gene network. The two gene ontology biological process terms (GO:0015979 and GO:0009765) and two MapMap sub-bins (1.3.4 and 1.1.1.1) that were significantly enriched in module Turquoise implicated that photosynthesis related pathways are likely essential for acidity. Overall this study for the first time provides not only important insight into the *Ma1*-mediated gene network controlling acidity in mature apple fruit of diverse genetic background, but also relevant clues for future biological validation, including the three putative regulators, MDP0000190273, MDP0000319170 and MDP0000140330.

## Supporting data

The data sets supporting the results of this article are available in the NCBI Sequence Read Archive (SRA) repository (http://www.ncbi.nlm.nih.gov/Traces/sra/) under accession SRX673837.

## Additional files

Additional file 1: Table S1. Fruit harvest date and weight. (DOCX 29 kb) Additional file 2: Table S2. Overview of RNA-seq reads mapping. (DOCX 31 kb) Additional file 3: Table S3. List of primers used in qRT-PCR. (DOCX 29 kb) Additional file 4: Table S4. List of the 1301 FAAGs and their RPKM values and assignment in WGCNA modules. (XLSX 550 kb) Additional file 5: Figure S1. Analysis of modules Black, Brown, Blue and Yellow. (A) Module eigengene values across the 29 samples, including 17 in *Ma*_ on left and 12 in *mama* on right. Samples are represented by the combination of a letter (abbreviated cultivar name) and a number (replicate) (see legends in Fig. 1, 4 for keys). (B) Correlation between module membership (MM) and gene significance (GS) for malate. (PPTX 75 kb) Additional file 6: Table S5. List of the most significant acidity genes (MSAGs). (DOCX 31 kb) Additional file 7: Table S6. List of intramodular hub genes in modules highly associated with acidity. (XLSX 26 kb) Additional file 8: Table S7. Regulator genes identified by Lemon-Tree. (DOCX 31 kb) Additional file 9: Figure S2. Regulator M190273 and its other five assigned tight clusters. Elements and their contents, formats and messages are same as those noted in Fig. 8a. (A) Cluster 8 of 31 genes. (B) Cluster 9 of 28 genes. (C) Cluster 40 of 55 genes. (D) Cluster 21 of 11 genes. (E) Cluster 45 of 10 genes. (PPTX 238 kb) Additional file 10: Figure S3. Other regulators from modules Turquoise and Brown and their assigned tight clusters. Elements and their contents, formats and messages are same as those noted in Fig. 8a. (A) Regulator M239684 and Cluster 41 of 68 genes. (B) Regulator M239684 and Cluster 5 of 14 genes. (C) Regulator M239684 and Cluster 7 of 14 genes. (D) Regulator M753318 and Cluster 23 of 11 genes. (E) Regulator M753318 and Cluster 32 of 11 genes. (F) Regulator M175481 and Cluster 2 of 16 genes. (G) Regulator M134341 and Cluster 42 of 12 genes. (PPTX 213 kb) Additional file 11: Table S8. Enriched gene ontology (GO) terms and MapMan (sub)-bins in WGCNA modules. (XLSX 16 kb)

## Abbreviations

ALMT1: Aluminum-activated Malate Transporter1
DEGs: Differentially expressed genes
FAAGs: Fruit acidity associated genes
FDR: False discovery rate
GD: Golden Delicious
*Ma1*: *Malic acid1*
MSAGs: Most significant acidity genes
QTL: Quantities trait locus
RNA-seq: mRNA sequencing
RPKM: Reads per kilobase of exon model per million mapped reads
TA: Titratable acidity
