# Supplementary material for: Uncovering co-expression gene network modules regulating fruit acidity in diverse apples
Source: BMC Genomics. 2015 Aug 16;16(1):612. doi: 10.1186/s12864-015-1816-6 (PMC4537561; doi:10.1186/s12864-015-1816-6)
Supplement: Additional file 1: Table S1. — Fruit harvest date and weight. (DOCX 29 kb) [file 12864_2015_1816_MOESM1_ESM.docx]

Table S1. Fruit harvest date and weight

| Variety Name | Genotype | Harvest date (M/D/Y) | Fruit weight at harvest (g) |
| --- | --- | --- | --- |
| Cox's Orange Pippin | *Mama* | 9/6/2012 | 95.1±10.2 |
| Empire | *MaMa* | 10/4/2012 | 123.5±8.8 |
| Fuji | *Mama* | 10/19/2012 | 92.4±9.7 |
| Granny Smith | *MaMa* | 10/30/2012 | 110.2±5.6 |
| Jonathan | *Mama* | 9/6/2012 | 71.6±5.3 |
| Rome Beauty Law | *Mama* | 10/12/2012 | 104.3±4.6 |
| Britegold | *mama* | 8/27/2012 | 154.6±9.8 |
| Novosibirski Sweet | *mama* | 8/27/2012 | 37.1±3.1 |
| PI323617 | *mama* | 8/27/2012 | 49±11.3 |
| Sweet Delicious | *mama* | 10/11/2012 | 181.3±9.3 |
