# Supplementary material for: Uncovering co-expression gene network modules regulating fruit acidity in diverse apples
Source: BMC Genomics. 2015 Aug 16;16(1):612. doi: 10.1186/s12864-015-1816-6 (PMC4537561; doi:10.1186/s12864-015-1816-6)
Supplement: Additional file 2: Table S2. — Overview of RNA-seq reads mapping. (DOCX 31 kb) [file 12864_2015_1816_MOESM2_ESM.docx]

Table S2 Overview of RNA-seq reads mapping

| Sample | Raw Reads | Clean and high quality reads | Total mapped reads | | Uniquely mapped reads | |
| --- | --- | --- | --- | --- | --- | --- |
|  |  |  | Count | Rate (%) | Count | Rate (%) |
| B_rep1 | 19,988,850 | 17,183,102 | 12,340,567 | 71.8 | 10,503,103 | 61.1 |
| B_rep2 | 18,390,359 | 15,967,900 | 11,632,057 | 72.9 | 9,919,597 | 62.1 |
| B_rep3 | 13,774,683 | 12,196,429 | 9,117,411 | 74.8 | 7,767,062 | 63.7 |
| C_rep1 | 10,999,347 | 5,879,487 | 3,912,693 | 66.6 | 3,301,275 | 56.2 |
| C_rep2 | 9,961,580 | 8,074,981 | 5,967,316 | 73.9 | 5,039,465 | 62.4 |
| C_rep3 | 13,850,408 | 9,205,655 | 6,456,968 | 70.1 | 5,464,889 | 59.4 |
| E_rep1 | 25,330,875 | 18,714,706 | 13,603,484 | 72.7 | 11,468,509 | 61.3 |
| E_rep2 | 17,735,691 | 13,502,334 | 9,800,294 | 72.6 | 8,306,995 | 61.5 |
| E_rep3 | 17,996,572 | 12,718,442 | 8,795,281 | 69.2 | 7,480,931 | 58.8 |
| F_rep1 | 20,616,175 | 18,058,659 | 13,612,585 | 75.4 | 11,583,516 | 64.1 |
| F_rep2 | 27,314,760 | 23,152,071 | 16,750,157 | 72.4 | 14,292,438 | 61.7 |
| F_rep3 | 16,001,675 | 13,584,614 | 9,962,392 | 73.3 | 8,500,115 | 62.6 |
| G_rep1† | 18,464,198 | 4,623,073 | 1,468,015 | 31.8 | 1,256,656 | 27.2 |
| G_rep2 | 21,853,897 | 17,803,842 | 13,198,907 | 74.1 | 11,163,504 | 62.7 |
| G_rep3 | 23,204,576 | 18,751,220 | 13,925,419 | 74.3 | 11,785,136 | 62.9 |
| J_rep1 | 20,946,545 | 14,983,663 | 10,293,758 | 68.7 | 8,791,641 | 58.7 |
| J_rep2 | 17,072,375 | 12,233,366 | 8,768,882 | 71.7 | 7,449,848 | 60.9 |
| J_rep3 | 30,600,975 | 20,564,326 | 14,385,729 | 70.0 | 12,204,083 | 59.4 |
| N_rep1 | 33,446,343 | 24,798,040 | 17,186,475 | 69.3 | 14,342,415 | 57.8 |
| N_rep2 | 26,028,574 | 19,793,799 | 13,983,011 | 70.6 | 11,647,295 | 58.8 |
| N_rep3 | 21,201,256 | 18,023,003 | 12,860,135 | 71.4 | 10,754,282 | 59.7 |
| P_rep1 | 16,246,019 | 10,330,620 | 7,240,565 | 70.1 | 6,177,537 | 59.8 |
| P_rep2 | 12,162,206 | 8,419,919 | 5,998,102 | 71.2 | 5,098,137 | 60.6 |
| P_rep3 | 12,564,429 | 9,702,172 | 7,007,993 | 72.2 | 5,940,964 | 61.2 |
| R_rep1 | 27,347,497 | 23,059,766 | 17,390,763 | 75.4 | 14,944,880 | 64.8 |
| R_rep2 | 23,186,447 | 18,755,675 | 13,969,235 | 74.5 | 11,967,862 | 63.8 |
| R_rep3 | 24,034,622 | 20,437,227 | 15,343,668 | 75.1 | 13,147,371 | 64.3 |
| S_rep1 | 25,150,325 | 19,455,000 | 14,224,196 | 73.1 | 12,041,432 | 61.9 |
| S_rep2 | 23,260,887 | 17,303,217 | 12,431,146 | 71.8 | 10,546,286 | 61.0 |
| S_rep3 | 26,868,746 | 15,590,001 | 10,015,804 | 64.3 | 8,516,885 | 54.6 |
| Total | 615,600,892 | 462,866,309 | 331,643,008 |  | 281,404,109 |  |
| Mean | 20,520,030 | 15,428,877 | 11,054,767 | 70.5 | 9,380,137 | 59.8 |
| SD | 5,808,998 | 5,329,973 | 4,069,156 | 7.8 | 3,451,209 | 6.6 |

†The mapping rate of G_rep1 is too low and this sample is not used in the downstream RNA-seq analysis.
