# Supplementary material for: Uncovering co-expression gene network modules regulating fruit acidity in diverse apples
Source: BMC Genomics. 2015 Aug 16;16(1):612. doi: 10.1186/s12864-015-1816-6 (PMC4537561; doi:10.1186/s12864-015-1816-6)
Supplement: Additional file 3: Table S3. — List of primers used in qRT-PCR. (DOCX 29 kb) [file 12864_2015_1816_MOESM3_ESM.docx]

| Table S3. List of primers used in qRT-PCR | |
| --- | --- |
| Primer ID | Primer Sequence (5’ to 3’) |
| 2F_M_219042 | CACCCAAACCTGATGAGAAGA |
| 2R_M_219042 | TGCTTCAACAGCTTCACTGG |
| 4F_M_651862 | ATTGACGATCAAGTGGAGCA |
| 4R_M_651862 | ATCTGGGACCGGACAACTC |
| 11F_M_190273 | GCACAAGATGGAATCCTGAAA |
| 11R_M_190273 | CAACCTTCTTCCTCCCTGAA |
| 13F_M_132720 | TCAGCTTGAGAGGGTGAATGT |
| 13R_M_132720 | TCACAATTCTCGGCCTCTTT |
| 14F_M_815327 | AATGAAGATCGTTGTGAAGGTG |
| 14R_M_815327 | GACGAGGTCGGCTTTACTTCT |
| 15F_M_163222 | TACCACCACTTTGCTCCACA |
| 15R_M_163222 | TCATTTCTCTCCCACGGATT |
| 16F_M_196894 | GCATCACGAAGAAGACGATG |
| 16R_M_196894 | TTCTTGCCGTGAATCAACAA |
| Ma1F_M_252114 | GTACTCCGACTTGGGCTTCA |
| Ma1R_M_252114 | ACATCTTTGAGCGGCACTTT |
| ActionF_EB136338 | GGCTGGATTTGCTGGTGATG |
| ActionR_EB136338 | TGCTCACTATGCCGTGCTCA |
