# Supplementary material for: Uncovering co-expression gene network modules regulating fruit acidity in diverse apples
Source: BMC Genomics. 2015 Aug 16;16(1):612. doi: 10.1186/s12864-015-1816-6 (PMC4537561; doi:10.1186/s12864-015-1816-6)
Supplement: Additional file 5: Figure S1. — Analysis of modules Black, Brown, Blue and Yellow. (A) Module eigengene values across the 29 samples, including 17 in Ma_ on left and 12 in mama on right. Samples are represented by the combination of a letter (abbreviated cultivar name) and a number (replicate) (see legends in Fig. 1, 4 for keys). (B) Correlation between module membership (MM) and gene significance (GS) for malate. (PPTX 75 kb) [file 12864_2015_1816_MOESM5_ESM.pptx]

## Slide 1
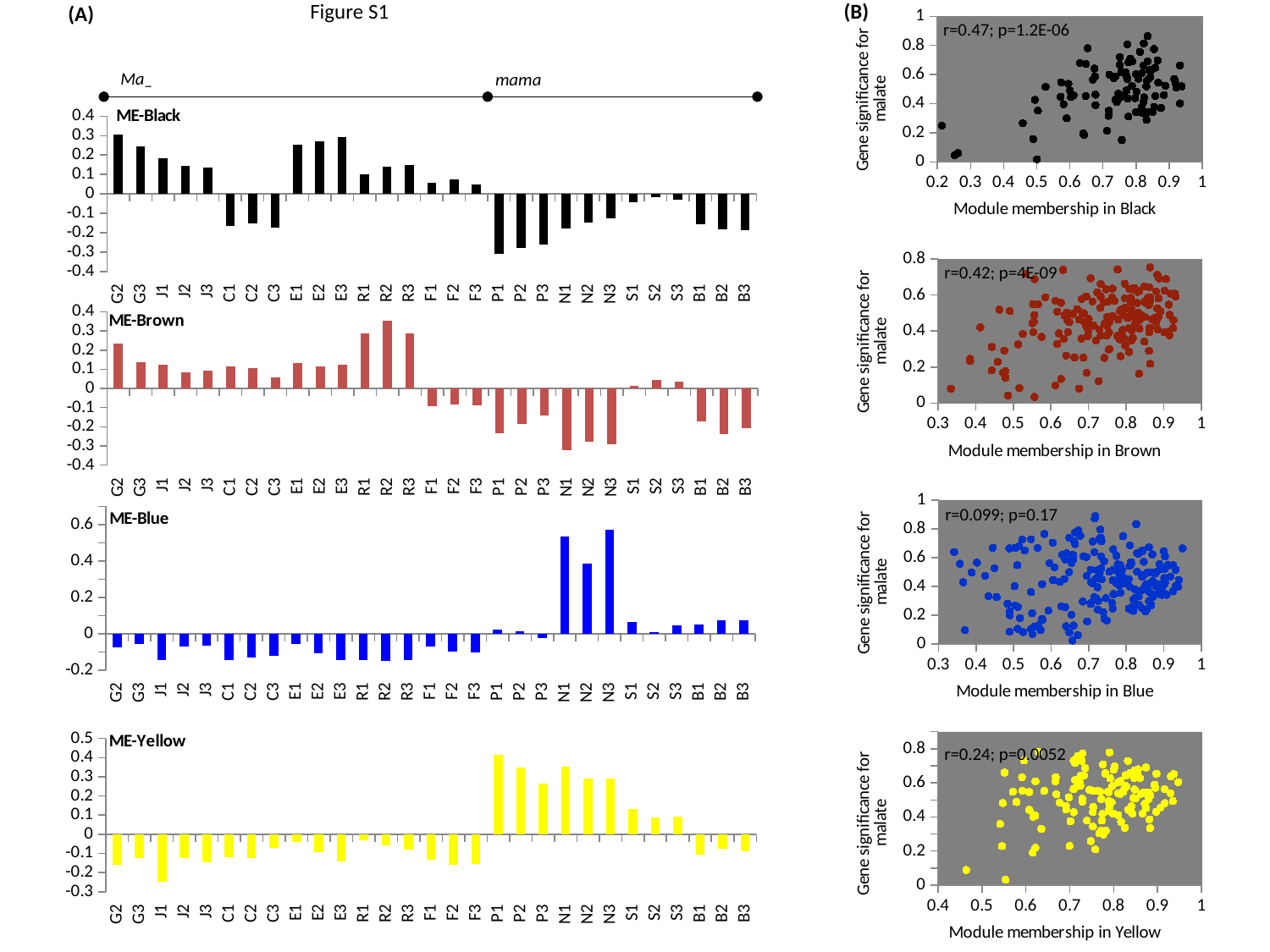

Figure S1
(B)
### Chart
| Category | ABS-MM.black |
|---|---|r=0.47; p=1.2E-06
### Chart
| Category | ABS-MM.brown |
|---|---|r=0.42; p=4E-09
### Chart
| Category | ABS-MM.blue |
|---|---|r=0.099; p=0.17
### Chart
| Category | ABS-MM.yellow |
|---|---|r=0.24; p=0.0052
(A)
### Chart: ME-Black
| Category | MEblack |
|---|---|
| G2 | 0.30786955 |
| G3 | 0.24520645 |
| J1 | 0.18139406 |
| J2 | 0.14615085 |
| J3 | 0.13608549 |
| C1 | -0.16555257 |
| C2 | -0.15140338 |
| C3 | -0.17228137 |
| E1 | 0.25417065 |
| E2 | 0.27225671 |
| E3 | 0.29270839 |
| R1 | 0.10061626 |
| R2 | 0.14026423 |
| R3 | 0.14768054 |
| F1 | 0.05616976 |
| F2 | 0.07634066 |
| F3 | 0.04648336 |
| P1 | -0.30789458 |
| P2 | -0.27823634 |
| P3 | -0.25999639 |
| N1 | -0.17622337 |
| N2 | -0.14751336 |
| N3 | -0.12624 |
| S1 | -0.04142297 |
| S2 | -0.01638533 |
| S3 | -0.03177418 |
| B1 | -0.15832027 |
| B2 | -0.18282717 |
| B3 | -0.18732566 |
### Chart: ME-Brown
| Category | MEblue |
|---|---|
| G2 | 0.23304015 |
| G3 | 0.13688496 |
| J1 | 0.1257116 |
| J2 | 0.0824304 |
| J3 | 0.09356397 |
| C1 | 0.11409799 |
| C2 | 0.10666424 |
| C3 | 0.05853981 |
| E1 | 0.13105118 |
| E2 | 0.11297639 |
| E3 | 0.124045 |
| R1 | 0.28755095 |
| R2 | 0.35334062 |
| R3 | 0.2855077 |
| F1 | -0.09362628 |
| F2 | -0.0832155 |
| F3 | -0.08700544 |
| P1 | -0.23398459 |
| P2 | -0.18380793 |
| P3 | -0.1398553 |
| N1 | -0.32352947 |
| N2 | -0.28029598 |
| N3 | -0.28992578 |
| S1 | 0.01476469 |
| S2 | 0.04353544 |
| S3 | 0.03421906 |
| B1 | -0.17325727 |
| B2 | -0.23995247 |
| B3 | -0.20946814 |
### Chart: ME-Blue
| Category | MEblack |
|---|---|
| G2 | -0.07617844 |
| G3 | -0.05821143 |
| J1 | -0.143347437 |
| J2 | -0.071843033 |
| J3 | -0.067392593 |
| C1 | -0.145962456 |
| C2 | -0.130833678 |
| C3 | -0.119608088 |
| E1 | -0.053682793 |
| E2 | -0.10747854 |
| E3 | -0.1440631 |
| R1 | -0.142463417 |
| R2 | -0.148123352 |
| R3 | -0.146232056 |
| F1 | -0.071121489 |
| F2 | -0.099261395 |
| F3 | -0.104696057 |
| P1 | 0.022290591 |
| P2 | 0.015798101 |
| P3 | -0.021406398 |
| N1 | 0.533147954 |
| N2 | 0.387147369 |
| N3 | 0.572559038 |
| S1 | 0.066740562 |
| S2 | 0.009761563 |
| S3 | 0.047466497 |
| B1 | 0.052725626 |
| B2 | 0.071873369 |
| B3 | 0.07239508 |
### Chart: ME-Yellow
| Category | MEblue |
|---|---|
| G2 | -0.15983633 |
| G3 | -0.12289694 |
| J1 | -0.24639592 |
| J2 | -0.12243063 |
| J3 | -0.14641029 |
| C1 | -0.12032306 |
| C2 | -0.12606583 |
| C3 | -0.06943559 |
| E1 | -0.04021251 |
| E2 | -0.09204163 |
| E3 | -0.14161369 |
| R1 | -0.03268435 |
| R2 | -0.05772241 |
| R3 | -0.07972796 |
| F1 | -0.13331762 |
| F2 | -0.16008806 |
| F3 | -0.15478928 |
| P1 | 0.41632109 |
| P2 | 0.34871822 |
| P3 | 0.26422336 |
| N1 | 0.35418374 |
| N2 | 0.29130248 |
| N3 | 0.2929788 |
| S1 | 0.13064169 |
| S2 | 0.08543777 |
| S3 | 0.0926753 |
| B1 | -0.10685431 |
| B2 | -0.07418941 |
| B3 | -0.08944661 |Ma_
mama
