# Supplementary material for: Uncovering co-expression gene network modules regulating fruit acidity in diverse apples
Source: BMC Genomics. 2015 Aug 16;16(1):612. doi: 10.1186/s12864-015-1816-6 (PMC4537561; doi:10.1186/s12864-015-1816-6)
Supplement: Additional file 6: Table S5. — List of the most significant acidity genes (MSAGs). (DOCX 31 kb) [file 12864_2015_1816_MOESM6_ESM.docx]

Table S5. List of the most significant acidity genes (MSAGs)

| Gene ID | WGCNA Module | Chromosomal locality and source contig | MapMan annotation | Gene significance for Malate |
| --- | --- | --- | --- | --- |
| G106959 | black | chr00_100502502..100505520_MDC020222.143 | 35.2_not assigned.unknown | 0.813 |
| M442350 | turquoise | Chr00_81138946_81139290-_MDC010433.118 | 35.2_not assigned.unknown, bromodomain-containing factor 1-like (NCBI) | 0.835 |
| M345601 | turquoise | Chr01_8240734_8240983+_MDC011411.282 | 35.2_not assigned.unknown | 0.868 |
| G104764 | turquoise | chr02_4648214..4657340_MDC012960.385 | 35.2_not assigned.unknown | 0.911 |
| G103681 | turquoise | chr02_4726897..4740003_MDC010364.126 | 35.2_not assigned.unknown | 0.847 |
| M364253 | turquoise | Chr03_4355237_4355555+_MDC008493.107 | 35.1_not assigned.no ontology, weakly similar to AT5G10860, CBS domain-containing protein | -0.808 |
| M282275 | black | Chr05_22396595_22398084+_MDC008530.144 | 34.16_transport.ABC transporters and multidrug resistance systems_ moderately similar to AT2G26910, PDR4, ATPDR4 | 0.807 |
| M230253 | turquoise | Chr06_23943904_23946141-_MDC006222.243 | 35.2_not assigned.unknown, weakly similar to AT3G17930 | 0.837 |
| M225641 | turquoise | Chr08_11400943_11406037+_MDC021050.213 | 10.6.2_cell wall.degradation.mannan-xylose-arabinose-fucose_ nearly identical to AT1G58370, ATXYN1, endo-1,4-beta-xylanase/ hydrolase | 0.833 |
| M800352 | turquoise | chr10_20385291_20386758+_MDC002048.388 | 1.1.1.2_PS.lightreaction.photosystem II.PSII polypeptide subunits_ weakly similar to AT1G79040, PSBR (photosystem II subunit R) | 0.833 |
| M525602 | turquoise | chr11_8118495_8121703-_MDC006029.332 | 30.2.11_signalling.receptor kinases.leucine rich repeat XI_ highly similar to AT3G47090, leucine-rich repeat transmembrane protein kinase, putative | 0.845 |
| M190273 | turquoise | chr14_19612925_19613476-_MDC010505.357 | 27.3.19_RNA.regulation of transcription.EIN3-like(EIL) transcription factor family_ weakly similar to AT3G20770, EIN3 | 0.854 |
| G202922 | turquoise | chr14_28527815..28549961_MDC020415.152 | 12.2.2_N-metabolism.ammonia metabolism.glutamine synthetase, weakly similar to Glutamine synthetase cytosolic isozyme (EC 6.3.1.2) | -0.807 |
| G104167 | blue | chr14_28919800..28944185_MDC011511.329 | 29.4_protein.postranslational modification_ weakly similar to at3g19420, PTEN 2 (PEN2) | 0.888 |
| M834327 | blue | chr14_28928711_28933207-_MDC011511.329 | 34.22_transport.cyclic nucleotide or calcium regulated channels_ highly similar to AT3G17700, ATCNGC20 | 0.871 |
| M651862 | blue | chr15_42799945_42801509+_MDC022899.263 | 29.4.1.57_protein.postranslational modification.kinase.receptor like cytoplasmatic kinase VII_ moderately similar to AT4G33430, BAK1 (BRI1-ASSOCIATED RECEPTOR KINASE) | 0.832 |
| M727725 | black | chr16_1312873_1315415+_MDC003939.79 | 8.1.1.2_TCA / org transformation.TCA.pyruvate DH.E2, moderately similar to AT3G25860, LTA2; dihydrolipoyllysine-residue acetyltransferase | -0.864 |
| Ma1 | turquoise | chr16_1354893_1357723+_MDC012995.82 | 34.8_transport.metabolite transporters at the envelope membrane_ highly similar to ( 589) AT1G25480, ALMT1 | 0.801 |
