# Supplementary material for: Uncovering co-expression gene network modules regulating fruit acidity in diverse apples
Source: BMC Genomics. 2015 Aug 16;16(1):612. doi: 10.1186/s12864-015-1816-6 (PMC4537561; doi:10.1186/s12864-015-1816-6)
Supplement: Additional file 8: Table S7. — Regulator genes identified by Lemon-Tree. (DOCX 31 kb) [file 12864_2015_1816_MOESM8_ESM.docx]

Table S7. Regulator genes identified by Lemon-Tree

| Regulator | WGCNA Module | Annotation | Gene significant for malate | Module member-ship | Cluster assigned | Prob. Score |
| --- | --- | --- | --- | --- | --- | --- |
| M190273 | Turquoise | EIN3-like(EIL) transcription factor family | 0.854 | 0.866 | 1 | 81.0 |
|  |  |  |  |  | 8 | 66.3 |
|  |  |  |  |  | 9 | 40.7 |
|  |  |  |  |  | 21 | 62.9 |
|  |  |  |  |  | 40 | 68.5 |
|  |  |  |  |  | 45 | 72.2 |
| M525602 | Turquoise | receptor kinases,leucine rich repeat XI | 0.845 | 0.884 | 22 | 37.8 |
| M319170 | Turquoise | IQD2 like protein, calcium signalling | 0.662 | 0.923 | 0 | 36.7 |
| M239684 | Turquoise | Homeobox transcription factor family | -0.530 | 0.724 | 5 | 35.5 |
|  |  |  |  |  | 7 | 46.0 |
|  |  |  |  |  | 41 | 62.8 |
| M134341 | Turquoise | GRAS transcription factor family | 0.395 | 0.707 | 42 | 54.6 |
| M753318 | Brown | EXO (EXORDIUM), signalling in sugar and nutrient physiology | 0.419 | 0.863 | 23 | 44.2 |
|  |  |  |  |  | 32 | 50.1 |
| M175481 | Brown | phototropic-responsive NPH3 family protein, light signalling | 0.688 | 0.557 | 2 | 41.5 |
| M304815 | Green | receptor kinases, leucine rich repeat XI | -0.573 | 0.834 | 14 | 60.5 |
|  |  |  |  |  | 20 | 44.5 |
| M517262 | Green | APETALA2/Ethylene-responsive element binding protein family | 0.482 | 0.755 | 34 | 37.7 |
| M241866 | Green | DNA-binding protein, putative | 0.405 | 0.861 | 46 | 43.5 |
| M250876 | Red | IAA11 \| IAA11 (INDOLE-3-ACETIC ACID INDUCIBLE 11), transcription factor | 0.505 | 0.697 | 3 | 40.2 |
| G100412 | Pink | APETALA2/Ethylene-responsive element binding protein family | -0.642 | 0.817 | 16 | 44.6 |
