# Supplementary material for: Uncovering co-expression gene network modules regulating fruit acidity in diverse apples
Source: BMC Genomics. 2015 Aug 16;16(1):612. doi: 10.1186/s12864-015-1816-6 (PMC4537561; doi:10.1186/s12864-015-1816-6)
Supplement: Additional file 9: Figure S2. — Regulator M190273 and its other five assigned tight clusters. Elements and their contents, formats and messages are same as those noted in Fig. 8a. (A) Cluster 8 of 31 genes. (B) Cluster 9 of 28 genes. (C) Cluster 40 of 55 genes. (D) Cluster 21 of 11 genes. (E) Cluster 45 of 10 genes. (PPTX 238 kb) [file 12864_2015_1816_MOESM9_ESM.pptx]

## Slide 1
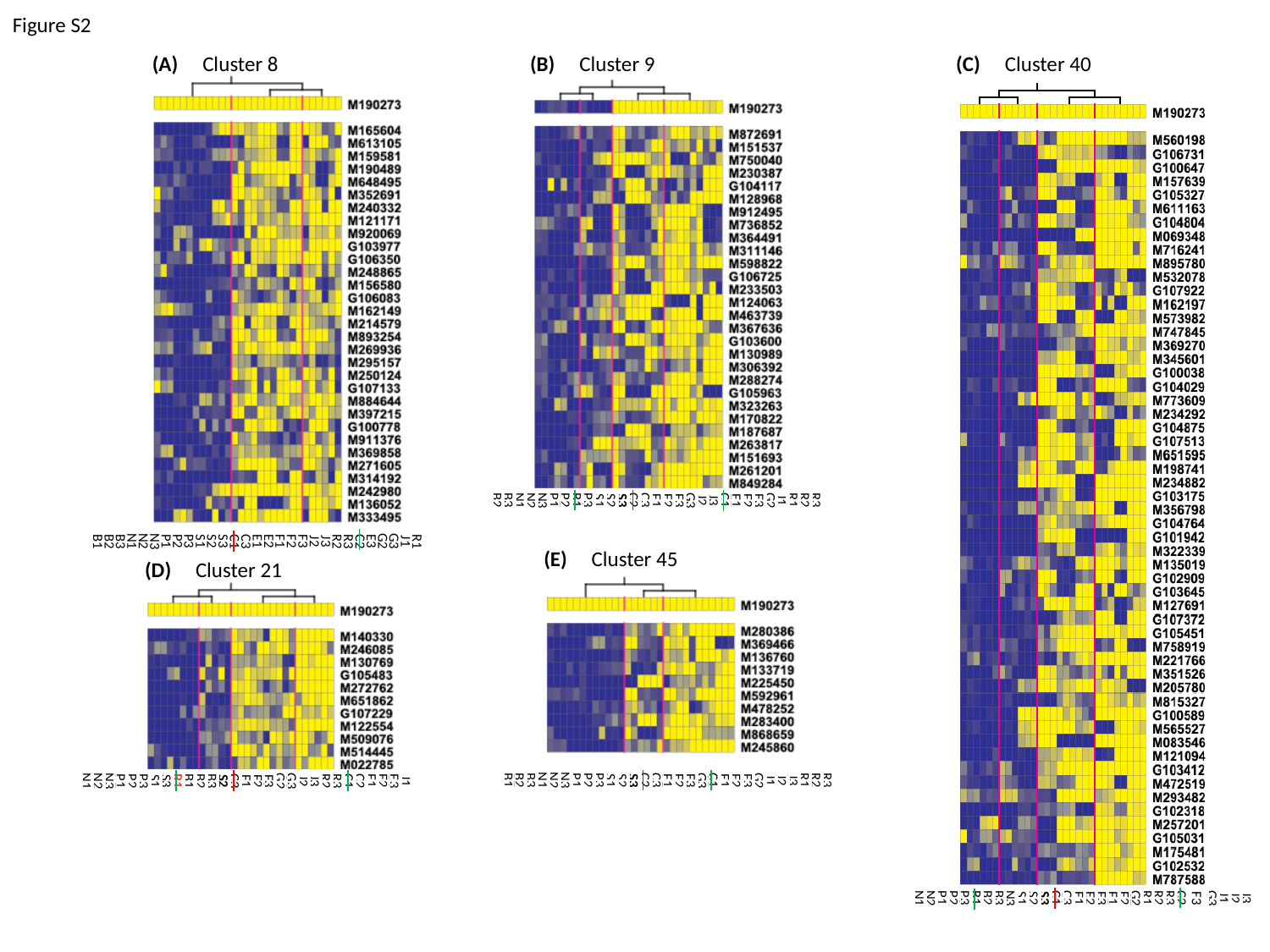

Figure S2
(A) Cluster 8
(B) Cluster 9
(C) Cluster 40
| B2 | B3 | N1 | N2 | N3 | P1 | P2 | B1 | P3 | S1 | S2 | S3 | C2 | C3 | F1 | F2 | F3 | G3 | J2 | J3 | C1 | E1 | E2 | E3 | G2 | J1 | R1 | R2 | R3 |
| --- | --- | --- | --- | --- | --- | --- | --- | --- | --- | --- | --- | --- | --- | --- | --- | --- | --- | --- | --- | --- | --- | --- | --- | --- | --- | --- | --- | --- |
| B1 | B2 | B3 | N1 | N2 | N3 | P1 | P2 | P3 | S1 | S2 | S3 | C1 | C3 | E1 | E2 | F1 | F2 | F3 | J2 | J3 | R2 | R3 | C2 | E3 | G2 | G3 | J1 | R1 |
| --- | --- | --- | --- | --- | --- | --- | --- | --- | --- | --- | --- | --- | --- | --- | --- | --- | --- | --- | --- | --- | --- | --- | --- | --- | --- | --- | --- | --- |
(E) Cluster 45
(D) Cluster 21
| B1 | B2 | B3 | N1 | N2 | N3 | P1 | P2 | P3 | S1 | S2 | S3 | C2 | C3 | F1 | F2 | F3 | G3 | C1 | E1 | E2 | E3 | G2 | J1 | J2 | J3 | R1 | R2 | R3 |
| --- | --- | --- | --- | --- | --- | --- | --- | --- | --- | --- | --- | --- | --- | --- | --- | --- | --- | --- | --- | --- | --- | --- | --- | --- | --- | --- | --- | --- |
| N1 | N2 | N3 | P1 | P2 | P3 | S1 | S3 | R1 | B1 | B2 | B3 | S2 | C3 | F1 | F2 | F3 | G2 | G3 | J2 | J3 | R2 | R3 | C1 | C2 | E1 | E2 | E3 | J1 |
| --- | --- | --- | --- | --- | --- | --- | --- | --- | --- | --- | --- | --- | --- | --- | --- | --- | --- | --- | --- | --- | --- | --- | --- | --- | --- | --- | --- | --- |
| N1 | N2 | P1 | P2 | P3 | B1 | B2 | B3 | N3 | S1 | S2 | S3 | C1 | C3 | E1 | E2 | E3 | F1 | F2 | G2 | R1 | R2 | R3 | C2 | F3 | G3 | J1 | J2 | J3 |
| --- | --- | --- | --- | --- | --- | --- | --- | --- | --- | --- | --- | --- | --- | --- | --- | --- | --- | --- | --- | --- | --- | --- | --- | --- | --- | --- | --- | --- |
