# Supplementary material for: Uncovering co-expression gene network modules regulating fruit acidity in diverse apples
Source: BMC Genomics. 2015 Aug 16;16(1):612. doi: 10.1186/s12864-015-1816-6 (PMC4537561; doi:10.1186/s12864-015-1816-6)
Supplement: Additional file 10: Figure S3. — Other regulators from modules Turquoise and Brown and their assigned tight clusters. Elements and their contents, formats and messages are same as those noted in Fig. 8a. (A) Regulator M239684 and Cluster 41 of 68 genes. (B) Regulator M239684 and Cluster 5 of 14 genes. (C) Regulator M239684 and Cluster 7 of 14 genes. (D) Regulator M753318 and Cluster 23 of 11 genes. (E) Regulator M753318 and Cluster 32 of 11 genes. (F) Regulator M175481 and Cluster 2 of 16 genes. (G) Regulator M134341 and Cluster 42 of 12 genes. (PPTX 213 kb) [file 12864_2015_1816_MOESM10_ESM.pptx]

## Slide 1
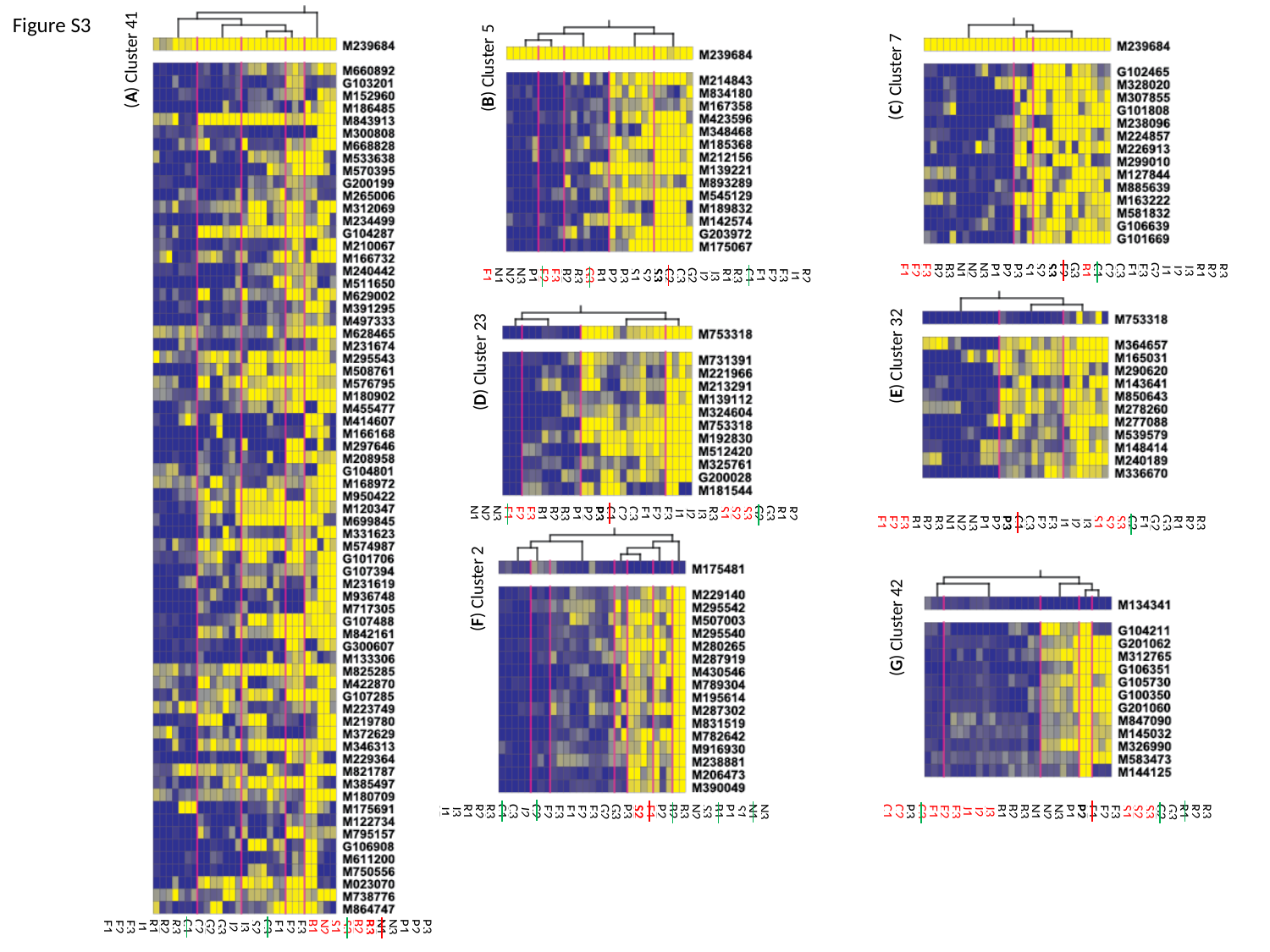

Figure S3
(A) Cluster 41
(B) Cluster 5
(C) Cluster 7
| F1 | F2 | F3 | B2 | B3 | N1 | N2 | N3 | P1 | P2 | P3 | S1 | S2 | S3 | E2 | G3 | B1 | C1 | C2 | C3 | E1 | E3 | G2 | J1 | J2 | J3 | R1 | R2 | R3 |
| --- | --- | --- | --- | --- | --- | --- | --- | --- | --- | --- | --- | --- | --- | --- | --- | --- | --- | --- | --- | --- | --- | --- | --- | --- | --- | --- | --- | --- |
| F1 | N1 | N2 | N3 | P1 | F2 | F3 | B2 | B3 | G3 | B1 | P2 | P3 | S1 | S2 | S3 | C2 | C3 | G2 | J2 | J3 | R1 | R3 | C1 | E1 | E2 | E3 | J1 | R2 |
| --- | --- | --- | --- | --- | --- | --- | --- | --- | --- | --- | --- | --- | --- | --- | --- | --- | --- | --- | --- | --- | --- | --- | --- | --- | --- | --- | --- | --- |
(E) Cluster 32
(D) Cluster 23
| N1 | N2 | N3 | F1 | F2 | F3 | B1 | B2 | B3 | P1 | P2 | P3 | C1 | C2 | C3 | E1 | E2 | E3 | J1 | J2 | J3 | R3 | S1 | S2 | S3 | G2 | G3 | R1 | R2 |
| --- | --- | --- | --- | --- | --- | --- | --- | --- | --- | --- | --- | --- | --- | --- | --- | --- | --- | --- | --- | --- | --- | --- | --- | --- | --- | --- | --- | --- |
| F1 | F2 | F3 | B1 | B2 | B3 | N1 | N2 | N3 | P1 | P2 | P3 | C1 | C3 | E2 | E3 | J1 | J2 | J3 | S1 | S2 | S3 | C2 | E1 | G2 | G3 | R1 | R2 | R3 |
| --- | --- | --- | --- | --- | --- | --- | --- | --- | --- | --- | --- | --- | --- | --- | --- | --- | --- | --- | --- | --- | --- | --- | --- | --- | --- | --- | --- | --- |
(F) Cluster 2
(G) Cluster 42
| J1 | J3 | R1 | R2 | R3 | C1 | C3 | J2 | C2 | E2 | E3 | F1 | F2 | F3 | G2 | G3 | P3 | S2 | E1 | P2 | B2 | B3 | N2 | S3 | B1 | P1 | S1 | N1 | N3 |
| --- | --- | --- | --- | --- | --- | --- | --- | --- | --- | --- | --- | --- | --- | --- | --- | --- | --- | --- | --- | --- | --- | --- | --- | --- | --- | --- | --- | --- |
| C1 | C2 | P3 | C3 | E1 | E2 | E3 | J1 | J2 | J3 | B1 | B2 | B3 | N1 | N2 | N3 | P1 | P2 | F1 | F2 | F3 | S1 | S2 | S3 | G2 | G3 | R1 | R2 | R3 |
| --- | --- | --- | --- | --- | --- | --- | --- | --- | --- | --- | --- | --- | --- | --- | --- | --- | --- | --- | --- | --- | --- | --- | --- | --- | --- | --- | --- | --- |
| E1 | E2 | E3 | J1 | R1 | R2 | R3 | C1 | C2 | G2 | G3 | J2 | J3 | S2 | C3 | F1 | F2 | F3 | B1 | N2 | S1 | S3 | B2 | B3 | N1 | N3 | P1 | P2 | P3 |
| --- | --- | --- | --- | --- | --- | --- | --- | --- | --- | --- | --- | --- | --- | --- | --- | --- | --- | --- | --- | --- | --- | --- | --- | --- | --- | --- | --- | --- |
